# Clinical significance for combined coagulation indexes in epithelial ovarian cancer prognosis

**DOI:** 10.1186/s13048-021-00858-1

**Published:** 2021-08-17

**Authors:** Jiani Yang, Yue Jin, Shanshan Cheng, Chao Wang, Nan Zhang, Shan Huang, Yaqian Zhao, Yu Wang

**Affiliations:** 1 Department of Obstetrics and Gynecology, Renji Hospital, School of Medicine, Shanghai Jiaotong University, Shanghai, China; 2 Shanghai Key Laboratory of Gynecologic Oncology, Shanghai, 200127 China

**Keywords:** Epithelial ovarian cancer, Prognostic value, Risk stratification, Coagulation system

## Abstract

**Background:**

Increasing evidence supported an association between cancer and coagulation system. We aimed to identify prognostic values of coagulation biomarkers in epithelial ovarian cancer (EOC).

**Methods:**

A retrospective study was conducted on patients who underwent optimal tumor debulking followed by platinum-based chemotherapy at our institution. The predictive value of coagulation variables was evaluated by receiver operating characteristic (ROC) curves. Through Cox hazards regression models, prognostic factors were determined for recurrence-free survival (RFS) and overall survival (OS). Survival curves were visualized by Kaplan–Meier method and compared through Log-rank analysis.

**Results:**

We involved 482 EOC patients and followed up for 64 (range, 36–87) months. According to ROC curves, D-dimer and International normalized ratio (INR) had superior predictive value than other coagulation indexes, with area under curve (AUC) of 0.758 and 0.742. Patients were then stratified into three combined D-dimer and INR (DD-INR) groups based on the cut-off value of 0.97 mg/L and 0.86, respectively. Through regression analysis, we demonstrated that age (HR 1.273; 95%CI 1.048–2.047; *p* = 0.045), pathological grade (HR 1.419; 95% CI 1.102–2.491; *p* = 0.032), clinical stage (HR 2.038; 95%CI 1.284–3.768; *p* = 0.008), CA-125 (HR 1.426; 95%CI 1.103–1.894; *p* = 0.038) and DD-INR (HR 2.412; 95%CI 1.683–3.241; *p* = 0.009) were independent prognostic factors. Survival analysis showed that patients with higher DD-INR experienced poor survival (*p* = 0.0013 for RFS and *p* = 0.0068 for OS). Further subgroup analysis revealed that evaluated DD-INR was significantly associated with poor survival among patients with advanced stage (*p* = 0.0028 for RFS and *p* = 0.0180 for OS).

**Conclusion:**

Our findings suggested that coagulation indexes, especially the combined DD-INR were promising biomarkers for prognosis stratification in EOC patients, especially those with advanced clinical stages.

## Background

Ovarian cancer (OC) is one of the most lethal gynecologic cancers, with 21,750 new cases and 13,940 deaths estimated for 2020 in the United States [1]. Owing to the lack of early physical signs and symptoms, almost 70% of OC cases were diagnosed at an advanced clinical stage, leading to a poor 5-year survival rate of 40% [2, 3]. Approximately 80% of epithelial ovarian cancer (EOC) patients suffered tumor progression or recurrence, despite development in therapy over past decades [4, 5]. To date, conventional tumor biomarkers, including CA-125, CA-199, and Human epididymis protein 4 (HE4) have been used to predict the survival of EOC patients with limited sensitivity and specificity [6]. Therefore, more effective biomarkers are of great urgency for risk stratification and individualized treatment in the realm of precise medicine [7].

Recently, increasing evidence showed that hyper-coagulability and fibrinolysis had a significant relationship with tumor development, progression, and dissemination [8]. As a degradation product crosslinked by activation factor XIII and hydrolyzed by fibrinolytic enzyme, D-dimer could serve as a biomarker for activated coagulation and fibrinolysis [9, 10]. Prothrombin time (PT), as a frequently used routine coagulation test, could be used to evaluate the “extrinsic coagulation pathway” [11]. To overcome the problem of marked variation for PT among different laboratories, International normalized ratio (INR) was introduced to standardize the PT value [12]. Moreover, previous studies showed that abnormal pre-treatment coagulation parameters, including D-dimer and INR, could predict poor survival in various malignancies, including colorectal, lung, and liver carcinoma [13–15].

However, as far as we know, studies assessing the prognostic value of the combination of plasma D-dimer and INR in EOC patients haven’t been reported yet. In this study, we aimed to examine the association between pre-treatment coagulation biomarkers and EOC prognosis, in order to provide cues for early intervention of EOC patients and support clinical decision-making. Specially, we stratified EOC patients into three groups based on DD-INR and further evaluated the prognostic application of this stratification method. We present the following article in accordance with the CONSORT reporting checklist.

## Results

### Patients clinicopathological characteristics

The overall clinicopathological features of EOC patients involved were listed in Table 1. A total of 482 EOC patients were finally involved according to the inclusion and exclusion criteria, with the mean age of 57.83 ± 5.93 years old. Based on International Federation of Obstetrics and Gynecology Association (FIGO) stage system, patients with early (FIGO I or II) and advanced (FIGO III or IV) clinical stages accounted for 167 (34.65%) and 315 (65.35%), respectively. There were 293 (60.79%) patients who presented with histology-proved serous subtype and 185 (38.38%) patients with low pathological grade (G1 or G2). The median and mean follow-up time for all patients was 64 months (range, 36–87 months) and 58.83 ± 23.94 months, respectively.

**Table 1:** Clinicopathological characteristics of epithelial ovarian cancer (EOC) patients

Variable	Total patients (*n* = 482)	DD-INR score			*p*-value
		**DD-INR = 0 (*n* = 139)**	**DD-INR = 1 (*n* = 178)**	**DD-INR = 2 (*n* = 165)**	
**Age (years)**	57.83 ± 5.93	57.32 ± 6.49	58.20 ± 5.31	57.92 ± 6.18	0.422
**BMI (kg/m2)**	23.12 ± 1.12	23.02 ± 1.03	22.98 ± 1.09	23.20 ± 1.12	0.144
**Menopausal status, n (%)**					0.629
** Pre/peri-menopause**	170 (35.27%)	52 (10.79%)	58 (12.03%)	60 (12.45%)	-
** Post-menopause**	312 (64.73%)	87 (18.05%)	120 (24.90%)	105 (21.78%)	-
**Tumor size (cm)**	6.21 ± 3.39	5.92 ± 3.87	6.32 ± 4.27	6.15 ± 3.24	0.426
**Pathological grade, n (%)**					0.015
** G1-2**	185 (38.38%)	52 (10.79%)	82 (17.01%)	51 (10.58%)	-
** G3**	297 (61.62%)	87 (18.05%)	96 (19.92%)	114 (23.65%)	-
**Clinical stage, n (%)**					0.003
** I-II**	167 (34.65%)	60 (12.45%)	66 (13.69%)	41 (8.51%)	-
** III-IV**	315 (65.35%)	79 (16.39%)	112 (23.24%)	124 (25.73%)	-
**Histological type, n (%)**					0.112
** Serous**	293 (60.79%)	87 (18.05%)	107 (22.20%)	99 (20.54%)	-
** Mucinous**	85 (17.63%)	29 (6.02%)	26 (5.39%)	30 (6.22%)	-
** Endometrioid**	57 (11.83%)	18 (3.73%)	21 (4.36%)	18 (3.73%)	-
** Others**	47 (9.75%)	5 (1.04%)	24 (4.98%)	18 (3.73%)	-
**Fibrinogen (g/L)**	4.25 ± 1.28	3.92 ± 0.89	4.03 ± 1.08	4.47 ± 1.35	0.021
**CA-125 (U/mL)**	989.62 ± 392.25	923.18 ± 383.84	997.34 ± 366.27	1040.73 ± 398.27	0.028
**CA-199 (U/mL)**	129.17 ± 58.73	130.48 ± 49.20	128.49 ± 56.32	126.49 ± 60.59	0.825
**HE4 (pmol/L)**	531.40 ± 89.04	534.23 ± 80.27	522.49 ± 69.03	540.73 ± 74.52	0.293
**APTT (s)**	32.74 ± 4.19	32.89 ± 3.72	31.82 ± 4.98	32.25 ± 5.02	0.129
**PT (s)**	11.43 ± 3.20	12.86 ± 2.94	11.57 ± 3.42	10.40 ± 2.36	< 0.001
**TT (s)**	14.72 ± 3.58	15.39 ± 4.02	14.92 ± 3.64	14.45 ± 3.27	0.081
**D-dimer (mg/L)**	0.64 ± 0.28	0.46 ± 0.23	0.78 ± 0.34	1.09 ± 0.25	< 0.001
**INR**	0.97 ± 0.35	1.13 ± 0.74	0.96 ± 0.48	0.75 ± 0.27	< 0.001
**Follow-up (months)**	58.83 ± 23.94	60.13 ± 29.85	59.01 ± 20.48	57.85 ± 19.26	0.694

*Abbreviation*: *BMI* body mass index, *DD-INR* the combination of D-dimer and International normalized ratio, *AFP* Alpha-fetoprotein, *CEA* Carcinoembryonic antigen, *HE4* Human epididymis protein 4, *APTT* activated partial thromboplastin time, *TT* thrombin time, *PT* prothrombin time

**Fig. 1:**
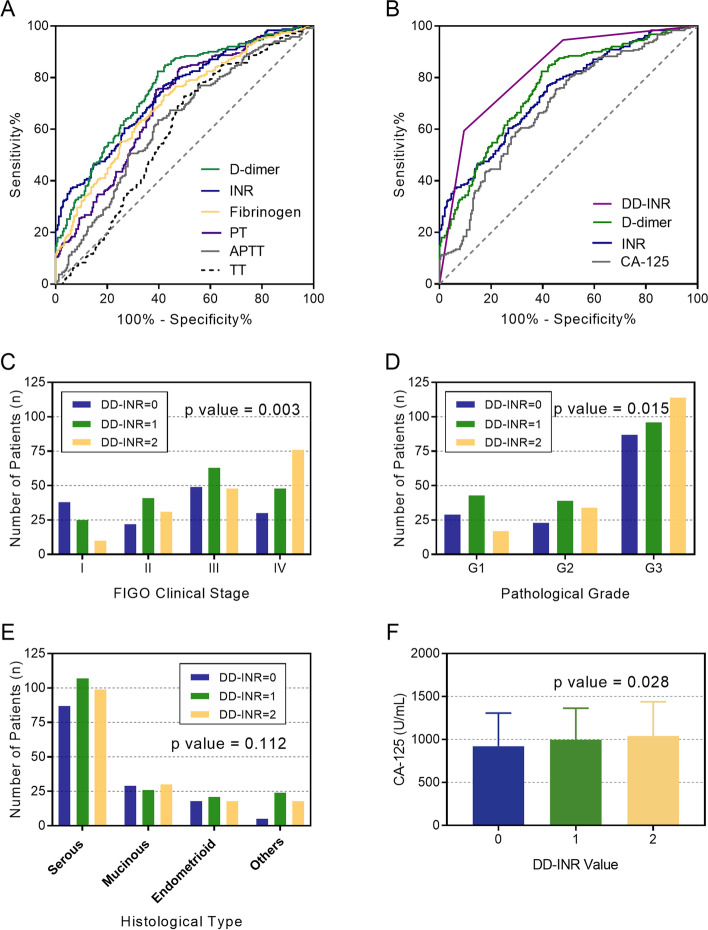
Receiver operating characteristic (ROC) curves for the predictive survival in epithelial ovarian cancer (EOC) patients by (**A**) coagulation system variables including D-dimer, international normalized ratio (INR), fibrinogen, prothrombin time (PT), activated partial thromboplastin time (APTT), and thrombin time (TT); **B** the combination of D-dimer and INR (DD-INR), compared with D-dimer, INR, and CA-125. Relationship between the combination score of D-dimer and International Normalized Ratio (DD-INR) and (**C**) the International Federation of Gynecology and Obstetrics (FIGO) clinical stage, **D** pathological grade, **E** histological type, and **F** CA-125 level

According to the Receiver operating characteristic (ROC) curves of coagulation variables, D-dimer and INR had superior predictive value for EOC survival compared to activated partial thromboplastin time (APTT), PT, thrombin time (TT), fibrinogen, and CA-125, with the area under curve (AUC) of 0.758 (95%CI 0.717–0.800) and 0.742 (95%CI 0.699–0.785), respectively (Fig. 1A and B). Based on the Youden index of ROC curves, the cut-off values were set at 0.97 mg/L for D-dimer and 0.86 for INR, for which sensitivity was determined as 82.4 and 76.7%, while specificity was determined as 60.4 and 58.3%. Patients were then classified into three DD-INR score groups referring to each cut-off value as follows: a DD-INR score of 2: with high D-dimer (> = 0.97 mg/L) and low INR (< 0.86), a DD-INR score of 1: with either of these coagulation abnormalities and a DD-INR score of 0: with neither of the abnormalities. The DD-INR score of all patients was allocated as follows: DD-INR = 0, 139 (28.84%) patients; DD-INR = 1, 178 (36.93%) patients; and DD-INR = 2, 165 (34.23%) patients. The AUC value for the DD-INR scoring system was 0.831 (95%CI 0.794–0.867) based on the ROC curve. Moreover, according to the exclusion criteria, we excluded 28 patients who were lost to follow-up, which might lead to selection bias. Considering that, we further evaluated the DD-INR score of these 28 patients: DD-INR = 0 (*n* = 7), DD-INR = 1 (*n* = 10), and DD-INR = 2 (*n* = 11). The results indicated that there was no significant difference of lost to follow-up rate among three DD-INR groups (*p* = 0.842).

### Correlation analysis between DD-INR score and clinicopathological factors

The correlation between preoperative DD-INR and other clinicopathological characteristics among all EOC patients was also shown in Table 1. We found that patients with higher DD-INR score tended to have more advanced FIGO stage (*p* = 0.003, Fig. 1C), higher pathological grade (*p* = 0.015, Fig. 1D). For histological subtypes, 87 (18.05%), 107 (22.20%) and 99 (20.54%) patients with DD-INR score of 0, 1, 2 were diagnosed as serous ovarian carcinoma (*p* = 0.112, Fig. 1E). Moreover, higher DD-INR was also significantly related to higher fibrinogen (*p* = 0.021), longer PT (*p* < 0.001), and higher CA-125 concentration (*p* = 0.028, Fig. 1F).

### Univariate and multivariate regression analyses for RFS and OS

To determine the independent prognostic factors for RFS, we performed both univariate and multivariate analyses (Table 2). The univariate analysis revealed that tumor size, pathological grade, clinical stage, neutrophil, lymphocyte, fibrinogen, CA-125, HE4, APTT, and DD-INR were significantly related to recurrence-free survival (RFS) (*p* < 0.05). These indexes were then involved in multivariate analysis, which demonstrated that tumor size (HR 1.382; 95%CI 1.194–1.857; *p* = 0.042), pathological grade (HR 1.385; 95%CI 1.272–1.988; *p* = 0.029), FIGO stage (HR 1.921; 95%CI 1.254–3.102; *p* = 0.015), fibrinogen (HR 1.297; 95%CI 1.106–1.924; *p* = 0.033), CA-125 (HR 1.398; 95%CI 1.082–1.653; *p* = 0.026) and DD-INR (HR 2.453; 95%CI 1.648–3.788; *p* = 0.009) were independent prognostic factors for RFS.

**Table 2:** Univariate and multivariate cox hazards survival analysis for recurrence-free survival (RFS) in epithelial ovarian cancer (EOC) patients

Variables	Univariate regression analysis		Multivariate regression analysis	
	**HR (95% CI)**	***P*-value**	**HR (95% CI)**	***P*-value**
**Age**	1.217(1.063–1.856)	0.037	1.138(0.956–1.573)	0.089
**BMI**	1.159(0.842–1.985)	0.251	-	-
**Tumor size**	1.246(1.059–1.725)	0.028	1.382(1.194–1.857)	0.042
**Pathological grade**				
** G1-2**	Reference	-	Reference	-
** G3**	1.483(1.162–2.058)	0.018	1.385(1.272–1.988)	0.029
**Clinical stage**				
** I-II**	Reference	-	Reference	-
** III-IV**	1.894(1.360–2.753)	0.009	1.921(1.254–3.102)	0.015
**Histological type**				
** Serous**	Reference	-	-	-
** Others**	1.093(0.849–1.382)	0.135	-	-
**Neutrophil**	1.241(1.075–1.793)	0.036	1.205(0.947–1.630)	0.132
**lymphocyte**	0.794(0.524–0.989)	0.032	0.837(0.463–1.024)	0.098
**Platelet**	1.084(0.937–1.421)	0.245	-	-
**Fibrinogen**	1.329(1.125–1.895)	0.014	1.297(1.106–1.924)	0.033
**CA-125**	1.420(1.193–1.614)	0.009	1.398(1.082–1.653)	0.026
**CA-199**	0.992(0.854–1.228)	0.236	-	-
**AFP**	1.362(0.937–1.468)	0.368	-	-
**CEA**	1.049(0.732–1.840)	0.493	-	-
**HE4**	1.372(1.004–1.738)	0.034	1.304(0.893–1.786)	0.103
**DD-INR group**				
** 0**	Reference	-	Reference	-
** 1**	1.463(1.192–1.847)	0.019	1.456(1.164–2.048)	0.021
** 2**	2.419(1.738–3.580)	0.002	2.453(1.648–3.788)	0.009
**APTT**	0.893(0.473–0.983)	0.037	0.837(0.452–1.284)	0.242
**TT**	0.982(0.748–1.240)	0.139	-	-

*Abbreviation*: *BMI* body mass index, *DD-INR* the combination of D-dimer and International normalized ratio, *AFP* Alpha-fetoprotein, *CEA* Carcinoembryonic antigen, *HE4* Human epididymis protein 4, *APTT* activated partial thromboplastin time, *TT* thrombin time, *HR* hazard ratio, *95% CI* 95% confidence interval

Furthermore, we analyzed independent prognostic factors for overall survival (OS) in Table 3. Through univariable regression model, we found that age, tumor size, pathological grade, clinical FIGO stage, neutrophil, fibrinogen, CA-125, CA-199, APTT and DD-INR were significantly related to OS (*p* < 0.05). The multivariate analysis involving these features revealed that age (HR, 1.273; 95% CI, 1.048–2.047; *p* = 0.045), pathological grade (HR, 1.419; 95% CI, 1.102–2.491; *p* = 0.032), clinical stage (HR, 2.038; 95% CI, 1.284–3.768; *p* = 0.008), CA-125 (HR, 1.426; 95% CI, 1.103–1.894; *p* = 0.038) and DD-INR (HR, 2.412; 95% CI, 1.683–3.241; *p* = 0.009) were independent prognostic factors for OS in EOC patients.

**Table 3:** Univariate and multivariate cox hazards survival analysis for overall survival (OS) in epithelial ovarian cancer (EOC) patients

Variables	Univariate regression analysis		Multivariate regression analysis	
	**HR (95% CI)**	***P*-value**	**HR (95% CI)**	***P*-value**
**Age**	1.327(1.089–1.948)	0.037	1.273(1.048–2.047)	0.045
**BMI**	1.291(0.894–1.473)	0.248	-	-
**Tumor size**	1.561(1.210–1.826)	0.028	1.534(0.987–1.932)	0.132
**Pathological grade**				
** G1-2**	Reference	-	Reference	-
** G3**	1.356(1.130–1.825)	0.019	1.419(1.102–2.491)	0.032
**Clinical stage**				
** I-II**	Reference	-	Reference	-
** III-IV**	1.938(1.420–3.487)	0.015	2.038(1.284–3.768)	0.008
**Histological type**				
** Serous**	Reference	-	-	-
** Others**	1.241(0.948–1.462)	0.183	-	-
**Neutrophil**	1.382(1.039–1.826)	0.046	1.276(0.983–1.850)	0.221
**lymphocyte**	0.892(0.652–1.024)	0.078	-	-
**Platelet**	1.352(0.846–1.745)	0.328	-	-
**Fibrinogen**	1.537(1.241–1.938)	0.025	1.420(0.995–2.018)	0.089
**CA-125**	1.432(1.129–1.726)	0.019	1.426(1.103–1.894)	0.038
**CA-199**	1.138(1.037–1.655)	0.043	1.123(0.913–1.748)	0.347
**AFP**	1.328(0.784–1.526)	0.361	-	-
**CEA**	1.241(0.848–1.657)	0.497	-	-
**HE4**	1.452(0.913–1.728)	0.142	-	-
**DD-INR group**				
** 0**	Reference	-	Reference	-
** 1**	1.653(1.210–1.923)	0.007	1.536(1.129–2.031)	0.013
** 2**	2.390(1.738–2.872)	0.001	2.412(1.683–3.241)	0.009
**APTT**	0.783(0.482–0.929)	0.047	0.820(0.421–1.532)	0.492
**TT**	0.803(0.562–1.118)	0.193	-	-

*Abbreviation*: *BMI* body mass index, *DD*-INR the combination of D-dimer and International normalized ratio, *AFP* Alpha-fetoprotein, *CEA* Carcinoembryonic antigen, *HE4* Human epididymis protein 4, *APTT* activated partial thromboplastin time, *TT* thrombin time, *HR* hazard ratio, *95% CI* 95% confidence interval

### Prognostic value of DD-INR in EOC stratification

**Fig. 2:**
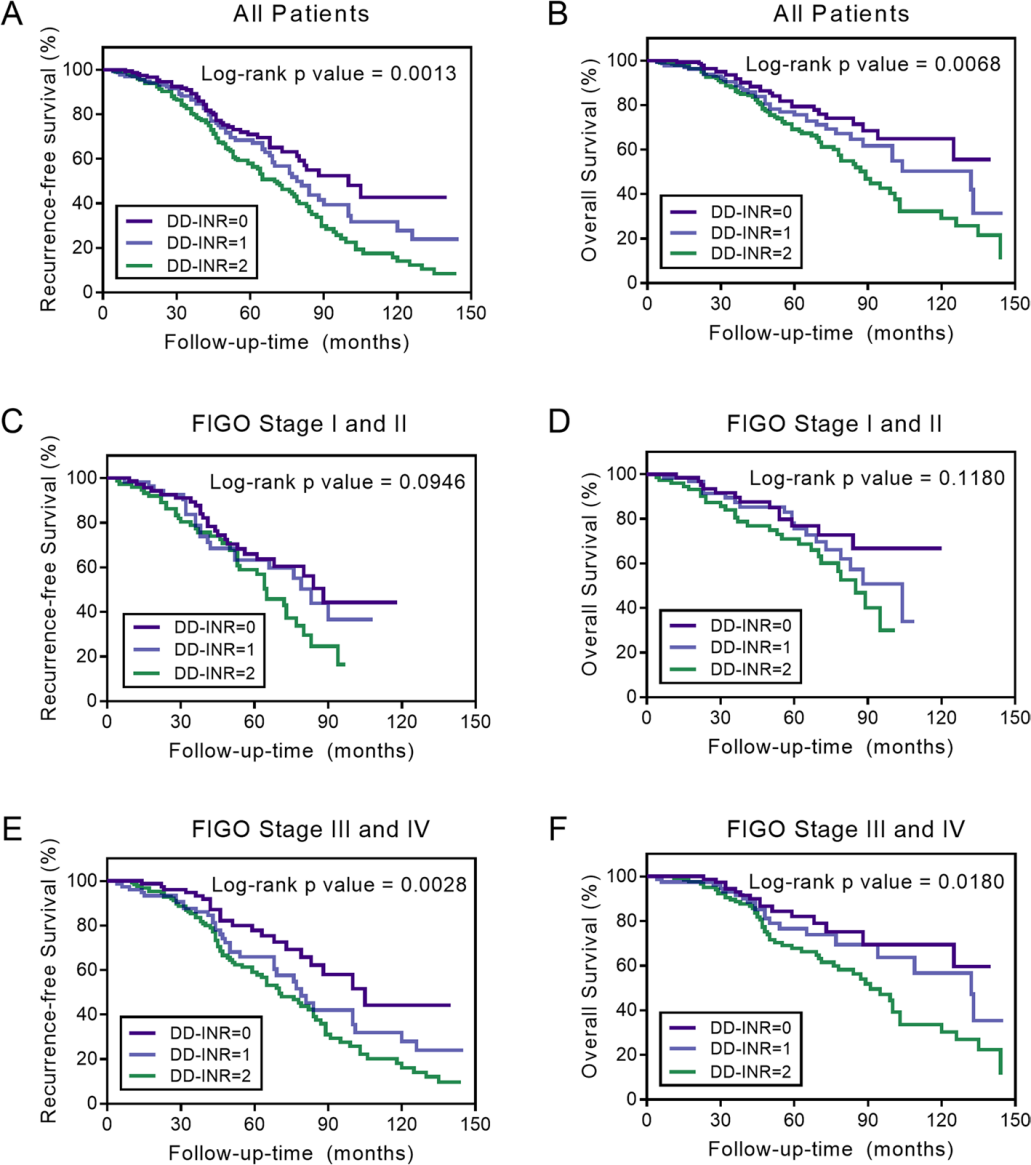
The Kaplan–Meier survival curves stratified by the combined score of D-dimer and International Normalized Ratio (DD-INR) for (**A**) recurrence-free-survival (RFS) among all patients; **B** overall survival (OS) among all patients; **C** RFS among patients with an early clinical stage (FIGO stage I and II); **D** OS among patients with early clinical stage; **E** RFS among patients with an advanced clinical stage (FIGO stage III and IV); and **F** OS among patients with advanced clinical stage

For all EOC patients, DD-INR score was significantly associated with RFS (log-rank, *p* = 0.0013, Fig. 2A) and OS (log-rank, *p* = 0.0068, Fig. 2B). The RFS rates among patients stratified by DD-INR were 39.57% (55/139), 23.60% (42/178), and 9.70% (16/165), respectively. The OS rates among patients stratified by DD-INR were 53.96% (75/139), 31.46% (56/178), and 13.33% (22/165). Results showed that higher DD-INR was significantly related to poorer RFS and OS (*p* < 0.005). We also revealed an association between DD-INR and survival among subgroups refer to FIGO clinical stage. For patients with an early clinical stage (FIGO stage I or II), DD-INR had no significant relationship to EOC prognosis, with the log-rank *p*-value of 0.0946 for RFS and 0.1180 for OS (Fig. 2C and D). Nevertheless, for patients with an advanced clinical stage (FIGO stage III or IV), DD-INR was significantly related to RFS (log-rank, *p* = 0.0028, Fig. 2E) and OS (log-rank, *p* = 0.0180, Fig. 2F).

## Discussion

Malignant tumor patients often suffer coagulation dysfunction, which manifested as a hyper-coagulable state activated by tumor-associated inflammatory cells [16]. In the present study, we estimated the prognostic value of coagulation biomarkers in EOC patients and concluded that the combination of preoperative D-dimer and INR was an independent prognostic factor for EOC. To the best of our knowledge, this is the first study evaluating the significance of the combined coagulation biomarker of DD-INR in EOC prognosis stratification.

In our study, we retrospectively collected clinical data from 482 EOC patients. Similar to previous observations [17, 18], we found a significant relationship between activation of coagulation system indicators, especially the evaluated INR and D-dimer, and poor prognosis in EOC patients.—For instance, a recent meta-analysis reported that high pre-treatment D-dimer concentration was an unfavorable prognosis factor for both progression-free survival (PFS) (HR 1.513; 95%CI 1.183–1.936; *P* = 0.001) and OS (HR 1.865; 95%CI, 1.469–2.367; *P* < 0.001), concluded from 5 cohort studies involving 813 small cell lung cancer (SCLC) patients [19]. As for OC, recently, Yamada and colleagues retrospectively conducted a multivariate analysis among 119 OC patients, which showed that a high pre-treatment plasma D-dimer level (≥ 1.0 µg/mL) was an independent risk factor for OC prognosis (*p* = 0.017), besides residual tumors (*p* < 0.001) and FIGO stage (*p* = 0.036) [20]. A meta-analysis included a total of 15 eligible studies with 1437 OC patients and concluded that elevated D-dimer concentration could predict increased risk of OC mortality (HR, 1.800; 95% CI, 1.283–2.523; *P* = 0.845) [21]. Up till now, several pieces of research have reported the significant relationship between high D-dimer concentration and venous thromboembolism (VTE) in patients with solid malignancies, which might be related to poor prognosis [22, 23]. However, as for OC, Haruki K and colleagues suggested plasma D-dimer as a prognosis predictor of OS in EOC patients, independently of venous thromboembolism and tumor extension [24]. Considering the controversy remains, in this research, we excluded those OC patients with concomitant diseases related to abnormal coagulation levels (such as pulmonary embolism, venous thromboembolism, and disseminated intravascular coagulation, etc.), which might be the confounding factors for us to explore the relationship between coagulation indexes and OC prognosis. As another important indicator of coagulation laboratories, the INR system has also been evaluated among cancer patients for its prognosis potentials. Previous studies showed that abnormal INR could predict poor survival in various malignancies, including colorectal, lung, and liver carcinoma [10–12]. For instance, Leyla Kilic and colleagues reported that elevated D-dimer and INR could indicate advanced disease stage in colorectal cancer (*P* = 0.03), proving their importance as surrogate biomarkers for cancer prognosis [15].

Up till now, emerging evidence have demonstrated the relationship between OC progression and the systematic coagulation system. On the one hand, in the complex process of OC progression, tumor cells can activate coagulation cascades by producing pro-coagulant proteins, inflammatory cytokines, and lipids, which subsequently leads to a hyper-coagulable status in vivo [25]. On the other hand, increased level of fibrinolysis and coagulation could become the soil for cancer growth, via secretion of cytokines and chemokines (including TNF, NF-KB, and macrophage inflammatory protein-1), as well as activation of macrophages [26]. Moreover, an activated coagulation state in tumor tissues could form a fibrin matrix to shield tumor cells against the immune attack and foster tumor growth, which finally contributes to poor cancer prognosis [27, 28]. Previous studies indicated that D-dimer, a degradation product produced by hydrolysis of fibrinolytic protein, could assess secondary fibrinolytic activity and hyper-coagulability [29]. Meanwhile, the INR system, applied to standardize PT, could evaluate the “extrinsic coagulation pathway” in individuals [30]. Considering that, both items are deemed as reliable indicators of coagulable status in vivo [31]. As a result, it is reasonable that high level of D-dimer and INR might be significantly correlated to OC prognosis, the main finding of our study. However, there is still an ongoing blank over detailed underlying mechanisms of the relationship between coagulation factors, especially D-dimer and INR, and tumor prognosis in OC patients; thus further in-depth insights are needed. Based on the ROC curves, we then divided patients into three groups by setting cut-off values (0.97 for D-dimer and 0.86 for INR). However, the INR cut-off value of 0.86 was within the traditional INR normal range of 0.8–1.5 [32]. Based on the ROC curve, we indicated that the cut-off value of 0.86 had the highest Youden index (sensitivity + specificity—1) for OC prognosis, with the sensitivity and specificity of 76.7 and 58.3%, respectively. Considering that the traditional INR normal range of 0.8–1.5 was applied to all populations to monitor anticoagulation status, it is reasonable that our cut-off value of 0.86 is different from the traditional normal range, since it was concluded from our 482 OC patients for tumor prognosis. Moreover, this problem might also be caused by the inherent limitations of retrospective study which carried out in a single institution, thus, multi-center studies with a larger database are of great urgency to validate the findings.

Stepwise, we found that the combination of DD-INR showed greater predictive value than D-dimer or INR alone, with higher specificity and sensitivity. We identified a significant association between DD-INR levels and several clinicopathological features, including pathological grade, clinical stage, fibrinogen, CA-125 concentration, and PT. Stepwise, univariate and multivariate analyses demonstrated that the preoperative DD-INR score was an independent prognosis factor for both RFS and OS among EOC patients. Besides DD-INR, other clinicopathological characteristics, including clinical stage, tumor grade, and tumor size, were also considered as promising prognosis factors for EOC survival, consistent with previous studies [18, 33]. However, since these features could only be obtained through invasive methods, blood biomarkers for predicting EOC prognosis are still of great importance [34]. The DD-INR scoring system, which is based on conventional blood examinations, could serve as a cost-effective and practical indicator with reliable prognostic value for EOC patients.

Nevertheless, the results revealed that preoperative DD-INR score could stratify patients into three risk categories of EOC recurrence and survival. Through further subgroup analysis refers to the clinical stage, we found that patients with higher DD-INR levels tended to have poor RFS and OS rates, especially for those with advanced FIGO stages. However, among early FIGO stage patients, there was no significant correlation between DD-INR and cancer prognosis. One possible explanation is that the compression of deep veins by solid tumor tissues or ascites is thought to promote coagulation in EOC, even in the early stage. Stepwise, various tumor-associated coagulation factors such as fibrin, tissue factors, and thrombin are implicated in mechanisms of tumor angiogenesis, growth, and metastasis, especially among advanced OC patients [28, 35]. A recent study also reported that plasma D-dimer level was significantly higher in advanced-stage (FIGO III and IV) OC patients (STD mean difference (SMD) 0.611, 95% CI 0.373–0.849, *P* = 0.442), than those in early stage (FIGO I and II), providing hints for the vital role of coagulation factors among OC patients with advanced FIGO stage [21]. However, since most OC cases were diagnosed at advanced stages, we involved 167/482 (34.65%) patients with early stages (FIGO I and II), among which only 41 individuals have 2 DD-INR points, might leading to a small sample bias. A meta-analysis indicated that activated coagulation state could predict increased risk of OC mortality (HR 1.800, 95%CI 1.283–2.523, *P* = 0.845), only based on researches with relatively large sample sizes (n > 100) [21]. Therefore, although our results suggested the DD-INR scoring system as an effective prognostic indicator for advanced EOC patients’ prognosis, further research with a more extensive database is of great urgency.’

However, there were still several limitations concerning this study. Firstly, we did not evaluate the variation of coagulation parameters during or after treatment. Therefore, we were limited to assess their relationship with cancer remission or progression. Secondly, we only enrolled patients who underwent an operation followed by platinum-based chemotherapy, resulting in selection bias. Moreover, the present retrospective study was carried out in a single institution. So, further prospective multi-center studies are still needed to support our findings.

## Conclusions

The combination of plasma D-dimer and INR could improve prognostic accuracy as a risk stratification criterion for EOC patients, indicating the importance of coagulation indexes in cancer recurrence and survival. Thus, the combined DD-INR scoring method might play an important role in therapy selection and disease monitoring to improve EOC patients’ survival in the upcoming future.

## Methods

### Patients selection

**Fig. 3:**
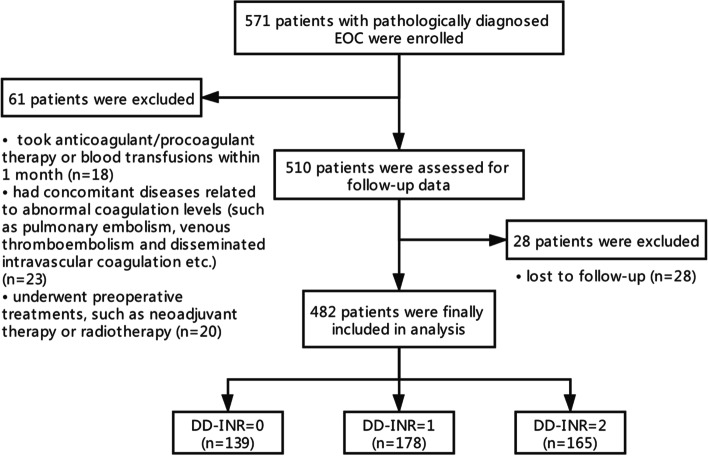
The flow chart for patient enrollment

We retrospectively reviewed data from 571 patients with pathologically diagnosed EOC at the Department of Obstetrics and Gynecology, Renji Hospital Affiliated to Shanghai Jiaotong University School of Medicine between May 2008 and December 2019. The inclusive criteria in this study were: (1) histologically confirmed EOC; (2) no co-existing or prior cancers within five years; (3) with detailed clinical, laboratory, imaging, and treatment data; and (4) underwent standard operation aimed to achieve optimal tumor debulking followed by platinum-based chemotherapy. Patients were excluded from our study if they: (1) took anticoagulant/pro-coagulant therapy or blood transfusions within one month (*n* = 18); (2) had concomitant diseases related to abnormal coagulation levels (such as pulmonary embolism, venous thromboembolism, and disseminated intravascular coagulation, etc.) (*n* = 23); (3) underwent preoperative treatments, such as neoadjuvant therapy or radiotherapy (*n* = 20); (4) were lost to follow-up (*n* = 28) (Fig. 3). Finally, 482 EOC patients were assessed in the analysis. This study was approved by the Ethics Committee of Renji Hospital Affiliated to Shanghai Jiaotong University School of Medicine. All patients provided informed consent for the usage of their data for research purposes.

### Data management

Clinicopathologic characteristics, including age, body mass index (BMI), comorbidities, menopausal status, tumor size, histology type, and tumor grade were collected retrospectively from the medical records. The clinical stage was defined based on the FIGO system. Routine blood tests and coagulation tests, including PT, APTT, TT, fibrinogen, D-dimer, and INR were conducted within three days before surgery. Of note, the value of D-dimer was quantified by operating procedures of the Innovance DD (SIEMENS assay), and expressed in DD units (μg/ml), with the normal value reference range of 0–0.5 μg/ml. Tumor biomarkers, including CA-125, CA-199, HE4, Carcinoembryonic antigen (CEA), and Alpha-fetoprotein (AFP) were also collected for analysis.

Moreover, to achieve optimal tumor debulking, all recruited patients underwent surgeries aiming at maximal ovarian tumor resection without visible residual tumor. Then, the surgery was followed by standardized paclitaxel and platinum chemotherapy. All involved patients with elevated D-dimer had vascular ultrasound and/or contrast-enhanced CT imaging to diagnose thrombosis. Patients were followed once every three months for two years, every six months for the next three years, and annually after that. Follow-ups were verified by checking clinical attendance records or telecommunicating with patients or their family members. The last follow-up was completed in October 2020. OS was defined from initial treatment to the last follow-up or death. RFS was identified from the initiation of therapy to the last follow-up or cancer recurrence, which was identified by the latest radiographic and clinical evidence.

### Statistical analysis

Continuous data were expressed as mean and standard deviation (mean ± SD) and compared using T-test. Category data were performed as numbers and percentages and analyzed by χ2 test. Through the ROC curve, the cut-off value of biomarkers was determined with the highest sensitivity and specificity. Univariate and multivariate analyses of clinicopathological variables were performed by the Cox hazards regression model. The survival curves were visualized using the Kaplan–Meier method and evaluated by the Log-rank test. *P*-value < 0.05 for a two-sided test was defined as statistically significant. All statistical tests were assessed with SPSS software (Version 23, IBM) and graphed through Prism Software (Version 7.0a, GraphPad).

## Data Availability

The datasets analyzed during the current study are available from the corresponding author on reasonable request.

## Abbreviations

EOC: Epithelial ovarian cancer
ROC: Receiver operating characteristic
RFS: Recurrence-free survival
OS: Overall survival
INR: International normalized ratio
AUC: Area under curve
HR: Hazard ratio
95%CI: 95% Confidence Interval
PT: Prothrombin time
BMI: Body mass index
FIGO: Federation of Obstetrics and Gynecology Association
APTT: Activated partial thromboplastin time
TT: Thrombin time
HE4: Human epididymis protein 4
CEA: Carcinoembryonic antigen
AFP: Alpha-fetoprotein
DD-INR: Combination of D-dimer and INR
VTE: Venous thromboembolism
SMD: STD mean difference
SCLC: Small cell lung cancer
PFS: Progression-free survival

## References

[1] Siegel RL, Miller KD, Jemal A (2020). Cancer statistics, 2020. CA Cancer J Clin.

[2] Ebell MH, Culp MB, Radke TJ (2016). A systematic review of symptoms for the diagnosis of ovarian cancer. Am J Prev Med.

[3] Bray F, Ferlay J, Soerjomataram I, Siegel RL, Torre LA, Jemal A (2018). Global cancer statistics 2018: GLOBOCAN estimates of incidence and mortality worldwide for 36 cancers in 185 countries. CA Cancer J Clin.

[4] Webb PM, Jordan SJ (2017). Epidemiology of epithelial ovarian cancer. Best Pract Res Clin Obstet Gynaecol.

[5] Jacobs IJ, Menon U, Ryan A, Gentry-Maharaj A, Burnell M, Kalsi JK, Amso NN, Apostolidou S, Benjamin E, Cruickshank D (2016). Ovarian cancer screening and mortality in the UK Collaborative Trial of Ovarian Cancer Screening (UKCTOCS): a randomised controlled trial. Lancet.

[6] Felder M, Kapur A, Gonzalez-Bosquet J, Horibata S, Heintz J, Albrecht R, Fass L, Kaur J, Hu K, Shojaei H (2014). MUC16 (CA125): tumor biomarker to cancer therapy, a work in progress. Mol Cancer.

[7] Arend R, Martinez A, Szul T, Birrer MJ (2019). Biomarkers in ovarian cancer: To be or not to be. Cancer.

[8] Wojtukiewicz MZ, Hempel D, Sierko E, Tucker SC, Honn KV (2016). Thrombin-unique coagulation system protein with multifaceted impacts on cancer and metastasis. Cancer Metastasis Rev.

[9] Johnson ED, Schell JC, Rodgers GM (2019). The D-dimer assay. Am J Hematol.

[10] Nam KW, Kim CK, Kim TJ, An SJ, Demchuk AM, Kim Y, Jung S, Han MK, Ko SB, Yoon BW (2017). D-dimer as a predictor of early neurologic deterioration in cryptogenic stroke with active cancer. Eur J Neurol.

[11] Tripodi A (2016). Thrombin generation assay and its application in the clinical laboratory. Clin Chem.

[12] Barcellona D, Fenu L, Marongiu F (2017). Point-of-care testing INR: an overview. Clin Chem Lab Med.

[13] Wang XP, Mao MJ, He ZL, Zhang L, Chi PD, Su JR, Dai SQ, Liu WL (2017). A retrospective discussion of the prognostic value of combining prothrombin time(PT) and fibrinogen(Fbg) in patients with Hepatocellular carcinoma. J Cancer.

[14] Tas F, Kilic L, Serilmez M, Keskin S, Sen F, Duranyildiz D (2013). Clinical and prognostic significance of coagulation assays in lung cancer. Respir Med.

[15] Kilic L, Yildiz I, Sen FK, Erdem MG, Serilmez M, Keskin S, Ciftci R, Karabulut S, Ordu C, Duranyildiz D (2015). D-dimer and international normalized ratio (INR) are correlated with tumor markers and disease stage in colorectal cancer patients. Cancer Biomark.

[16] Koizume S, Miyagi Y (2017). Potential coagulation factor-driven pro-inflammatory responses in ovarian cancer tissues associated with insufficient O(2) and plasma supply. Int J Mol Sci.

[17] Liu P, Wang Y, Tong L, Xu Y, Zhang W, Guo Z, Ni H (2015). Elevated preoperative plasma D-dimer level is a useful predictor of chemoresistance and poor disease outcome for serous ovarian cancer patients. Cancer Chemother Pharmacol.

[18] Luo Y, Kim HS, Kim M, Lee M, Song YS (2017). Elevated plasma fibrinogen levels and prognosis of epithelial ovarian cancer: a cohort study and meta-analysis. J Gynecol Oncol.

[19] Deng HY, Ma XS, Zhou J, Wang RL, Jiang R, Qiu XM (2021). High pretreatment D-dimer level is an independent unfavorable prognostic factor of small cell lung cancer: A systematic review and meta-analysis. Medicine (Baltimore).

[20] Yamada Y, Kawaguchi R, Iwai K, Niiro E, Morioka S, Tanase Y, Kobayashi H (2020). Preoperative plasma D-dimer level is a useful prognostic marker in ovarian cancer. J Obstet Gynaecol.

[21] Wu J, Fu Z, Liu G, Xu P, Xu J, Jia X (2017). Clinical significance of plasma D-dimer in ovarian cancer: A meta-analysis. Medicine (Baltimore).

[22] Huisman MV, Barco S, Cannegieter SC, Le Gal G, Konstantinides SV, Reitsma PH, Rodger M, VonkNoordegraaf A, Klok FA (2018). Pulmonary embolism. Nat Rev Dis Primers.

[23] Cushman M, Folsom AR, Wang L, Aleksic N, Rosamond WD, Tracy RP, Heckbert SR (2003). Fibrin fragment D-dimer and the risk of future venous thrombosis. Blood.

[24] Sakurai M, Satoh T, Matsumoto K, Michikami H, Nakamura Y, Nakao S, Ochi H, Onuki M, Minaguchi T, Yoshikawa H (2015). High pretreatment plasma D-dimer levels are associated with poor prognosis in patients with ovarian cancer independently of venous thromboembolism and tumor extension. Int J Gynecol Cancer.

[25] Falanga A, Panova-Noeva M, Russo L (2009). Procoagulant mechanisms in tumour cells. Best Pract Res Clin Haematol.

[26] Jensen T, Kierulf P, Sandset PM, Klingenberg O, Joo GB, Godal HC, Skjonsberg OH (2007). Fibrinogen and fibrin induce synthesis of proinflammatory cytokines from isolated peripheral blood mononuclear cells. Thromb Haemost.

[27] Fernandes CJ, Morinaga LTK, Alves JL, Castro MA, Calderaro D, Jardim CVP, Souza R (2019). Cancer-associated thrombosis: the when, how and why. Eur Respir Rev.

[28] Falanga A, Marchetti M, Vignoli A (2013). Coagulation and cancer: biological and clinical aspects. J Thromb Haemost.

[29] Duffett L, Castellucci LA, Forgie MA (2020). Pulmonary embolism: update on management and controversies. BMJ.

[30] Sallah S, Husain A, Sigounas V, Wan J, Turturro F, Sigounas G, Nguyen NP (2004). Plasma coagulation markers in patients with solid tumors and venous thromboembolic disease receiving oral anticoagulation therapy. Clin Cancer Res.

[31] Lin Y, Liu Z, Qiu Y, Zhang J, Wu H, Liang R, Chen G, Qin G, Li Y, Zou D (2018). Clinical significance of plasma D-dimer and fibrinogen in digestive cancer: A systematic review and meta-analysis. Eur J Surg Oncol.

[32] Kim HK, Hong KH, Toh CH, Scientific, Standardization Committee on DICoTISoT, Haemostasis (2010). Application of the international normalized ratio in the scoring system for disseminated intravascular coagulation. J Thromb Haemost.

[33] Xiang J, Zhou L, Li X, Bao W, Chen T, Xi X, He Y, Wan X (2017). Preoperative monocyte-to-lymphocyte ratio in peripheral blood predicts stages, metastasis, and histological grades in patients with ovarian cancer. Transl Oncol.

[34] Lisio MA, Fu L, Goyeneche A, Gao ZH, Telleria C (2019). High-grade serous ovarian cancer: basic sciences, clinical and therapeutic standpoints. Int J Mol Sci.

[35] Swier N, Versteeg HH (2017). Reciprocal links between venous thromboembolism, coagulation factors and ovarian cancer progression. Thromb Res.

